# Elements and Performance Indicators of Integrated Healthcare Programmes on Chronic Diseases in Six Countries in the Asia-Pacific Region: A Scoping Review

**DOI:** 10.5334/ijic.5439

**Published:** 2021-02-08

**Authors:** Kamilla Anna Pinter, Hanwen Zhang, Chang Liu, Bach Tran, Maulik Chokshi, Don Eliseo Lucerno-Prisno, Vikash Sharma, Shenglan Tang

**Affiliations:** 1 Duke Kunshan University, US; 2 ACCESS Health International, US; 3 Mainland China, Hong Kong and Singapore, ACCESS Health International, CN; 4 Hanoi Medical University, Johns Hopkins Bloomberg School of Public Health, VN; 5 London School of Hygiene and Tropical Medicine, UK; 6 Philippines Open University, PH; 7 Internal Medicine, School of Medical Sciences, College of Medicine, Nursing and Health Sciences, Fiji National University, FJ; 8 Medicine and Global Health, Duke Medical School/Duke Global Health Institute, US; 9 Global Health at SingHealth-Duke-NUS Global Health Institute, US

**Keywords:** integrated healthcare, integrated care delivery, chronic diseases, elements, performance

## Abstract

**Background and Aims:**

Globally, hospital-based healthcare models targeting acute care, are not effective in addressing chronic conditions. Integrated care programmes for chronic diseases have been widely developed and implemented in Europe and North America and to a much lesser extent in the Asia-Pacific region to meet such challenges. We completed a scoping review aiming to examine the elements of programmes identified in the literature from select study countries in the Asia-Pacific, and discuss important facilitators and barriers for design and implementation.

**Methods:**

The study design adopted a scoping review approach. Integrated care programmes in the study countries were searched in electronic databases using a developed search strategy and key words. Elements of care integration, barriers and facilitators were identified and charted following the Chronic Care Model (CCM).

**Results:**

Overall the study found a total of 87 integrated care programmes for chronic diseases in all countries, with 44 in China, 21 in Singapore, 12 in India, 5 in Vietnam, 4 in the Philippines and 1 in Fiji. Financial incentives were found to play a crucial role in facilitating integrated care and ensuring the sustainability of programmes. In many cases, the performance of programmes was found not to have been adequately assessed.

**Conclusion:**

Integrated care is important for addressing the challenges surrounding the delivery of long-term care and there is an increasing trend of integrated care programmes for chronic diseases in the Asia-Pacific. Evaluating the performance of integrated care programmes is crucial for developing strategies for implementing future programmes and improving already existing programmes.

## Background

According to the WHO in 2014, up to two thirds of government healthcare expenditure is spent on hospitals and approximately 300 million USD is lost per year due to hospital related inefficiencies globally [1]. However, in 2017, the World Bank and WHO estimated that half of the world’s population lack access to essential healthcare globally [2]. In the Asia Pacific region, the number of people over the age of 60 is expected to rise to 1.3 billion by 2050, due to declining fertility and increasing longevity [3]. Along with the aging of populations, WHO predicted a 20% increase in non-communicable disease (NCD) deaths in the Asia-Pacific between 2010 and 2020, which is higher than the 15% increase predicted in some other regions [4].

Currently, many health systems focus on hospital based acute care and disease treatment which does not adequately address the emerging challenges presented by ageing populations, increasing burden of NCDs, multi-morbidities and increasingly unhealthy lifestyles, all driving rising healthcare costs and compromised healthcare quality [2,5,6]. The persistence and complex nature of these issues calls for a comprehensive response over a sustained period of time and this is not easily delivered by hospital models of care focusing on acute issues and single episodes of illness [5,7]. While hospitals remain essential for delivering acute care, healthcare delivery reforms are needed to ensure the continuity of care across primary, hospital and post-acute settings [1,7]. There is clearly a need for long-term care in this region, and integrated care can have an important role [8].

Integrated care tackles fragmentation of care and also seeks to relieve the economic burden associated with long-term care and improve the quality and efficiency of service delivery [9,10]. As patients receive care from many different providers often across fragmented settings and institutions, their needs are not adequately met. Integrated care reduces this fragmentation through linking and coordinating services [10]. Up until now, efforts for integrating healthcare have been most extensively undertaken in North America and Europe [9]. Driven by the acknowledged benefits, countries in the Asia Pacific region have also implemented and piloted integrated care models but to a much lesser extent [5,9].

This scoping review will chart, present, and discuss integrated care programmes for chronic diseases based on extensive review of the literature for six select countries in the Asia-Pacific region including: China, Fiji, India, the Philippines, Singapore and Vietnam. The facilitators and barriers for integrated care programmes identified in the literature will be presented and key indicators for evaluation will be identified. To our knowledge, there is no review of integrated care for chronic diseases in these countries and reporting such information would be useful for the purpose of identifying facilitators and barriers and informing integrated care programmes undertaken in this region in the future. The questions the scoping review asked include the following:

- What kind of integrated care programmes/models for chronic diseases are being developed in the study countries?
- What key elements were included in these programmes/models?
- What were the facilitators and barriers associated with implementation, and performance indicators of these integrated care programmes/models for chronic diseases?

## Methods

### Study Design

The research team conducted a scoping review, using the PRISMA extension for Scoping Reviews (PRISMA-ScR) to guide reporting. A protocol was prepared and revised with comments and inputs from our key stakeholders.

### Definitions of Integrated Care

There is currently no official or single definition for integrated care. The concept may change with differing views, perspectives and expectations of various stakeholders such as patients, providers, communities, managers etc. across various locations. However, the WHO defines integrated health services as:

“health services that are managed and delivered so that people receive a continuum of health promotion, disease prevention, diagnosis, treatment, disease-management, rehabilitation and palliative care services, coordinated across the different levels and sites of care within and beyond the health sector, and according to their needs throughout the life course” [6, p.2].

Although the concept of integrated care is not uniformly defined and alternative terminologies such as “coordinated care”, “collaborative care”, “managed care”, “patient-centered care”, “continuity of care” and others may be used interchangeably, the common goal for all is improving health outcomes of populations with complex and chronic conditions. The closely related term, coordinated care, refers to the integration of medical and social service providers via the planning and implementation of a tailored plan [12]. Another term, patient-centered care, considers the individual’s specific health needs throughout the entire care process and encourages shared decision making between the patients, providers as well as family members [13].

### Data Sources and Search

Integrated care programmes for chronic diseases were selected from Singapore, China, the Philippines, India, Vietnam and Fiji. These countries have been selected to represent a good mix of lower-middle income (the Philippines, India, Vietnam and Fiji), upper-middle income (China) and high-income (Singapore) conditions based on the latest World Bank classification of economies [14].

The research team searched electronic databases including PubMed, Cochrane Library, Web of Science, EMBASE and Medline for publications between January 1, 2010 to June 30, 2018. Key search terms included “integrat* care”, “integrat* healthcare”, “integrat* health care”, “integrat* health service”, and “integrat* health delivery”. For all the databases, Boolean terms AND and OR were used to extract relevant studies. The full search strategy for PubMed is presented in ***Table 1***, and the other database searches are available from the corresponding author upon request.

**Table 1:** Full search strategy for PubMed.


DATABASE	PUBMED

Time coverage	From January 1, 2010 to June 30, 2018

Date of search	August 20, 2018

Limits	In: “Article Title, Abstract, Keywords” Date range: All years

Search query	((integrat* care) OR (integrat* healthcare) OR (integrat* health care) OR (integrat* health service) OR (integrat* health delivery)) AND ((Singapore) OR (China) OR (Philippines) OR (India) OR (Vietnam) OR (Fiji))


### Study Selection

There were three stages in the study selection process. In the first stage, all duplicates were removed. Inthe second stage, titles and abstracts were screened to identify papers for full-text screening. In the third stage, the selected papers were reviewed in full in accordance with inclusion and exclusion criteria. The second and third stages were performed by six reviewers. For each study country, two reviewers independently screened titles and abstracts, and examined full-text articles for eligibility. Disagreements between reviewers were resolved by a third independent reviewer.

Papers were included if:

- Any of the elements of integrated care (including multidisciplinary team, care coordinator/case manager, information sharing system, risk stratification, referral system, defined eligibility criteria, single point of patient referral, single assessment, formulation of health plan, use of telehealth, engaging users, self-management support and support of informal carers) were described in the text;
- Target population of the programme was patients with chronic conditions (according to the definition of the U.S. National Center for Health Statistics, chronic conditions refers to conditions that last 3 months or more, and require ongoing medical attention) requiring more than a single care episode;
- The location of the programme described is any of the six Asia-Pacific countries including Singapore, China, India, Vietnam, Philippines and Fiji.

Papers were excluded if:

- They were literature reviews of integrated healthcare models;
- No full text was available;
- The paper was not written in English.

### Data Abstraction

Data was also independently extracted by two reviewers for each study country focusing on the following:

- Literature Characteristics: title of the paper, year of publication, study type (ie. Randomized Control Trial, observational study etc.);
- Description of integrated care programme: name of programme, description, objective, context, element of integration and facilitators and barriers;
- Indicators of the programme: user and professional experience, care outcomes, utilization of services and cost-effectiveness.

### Data Charting and Collation

We conducted a structured charting and categorization of the selected programmes based on their characteristics and elements. We listed and described the differences in study and programme characteristics, elements of integrated care delivery, financial and non-financial incentives and performance of programmes by study country.

The elements of care integration were charted using the Chronic Care Model (CCM) proposed by Wagner et al (1996) which has been a popular guideline for integrated healthcare for chronic diseases. The CCM identifies six key elements of comprehensive integrated care programmes including self-management support, delivery system design, decision support, clinical information systems, the healthcare system, and community resources and policies [15]. Due to the limited availability of data from the literature we included in our review, we have charted the elements of integration for all the programmes, by delivery system design, self-management support and clinical information systems.

Enabling factors and incentives, including financial and non-financial were collated and discussed separately according to the target of the incentives ie. Service providers, service users, or the government. Incentives also included those related to systemic integration or the rules and policies which facilitate integrated care. Indicators related to performance of integrated care were grouped according to Donabedian’s framework for healthcare quality, consisting of structure, process and outcome [16]. “Structure” is defined as the setting, qualifications of healthcare providers and administration system for delivering healthcare. “Process” is the components of the healthcare delivered and “outcome” is the recovery, restoration and survival of the patient population [17]. Barriers, both financial and non-financial, were identified and discussed to inform future integrated care programmes.

## Results

### Literature search

**Figure 1:**
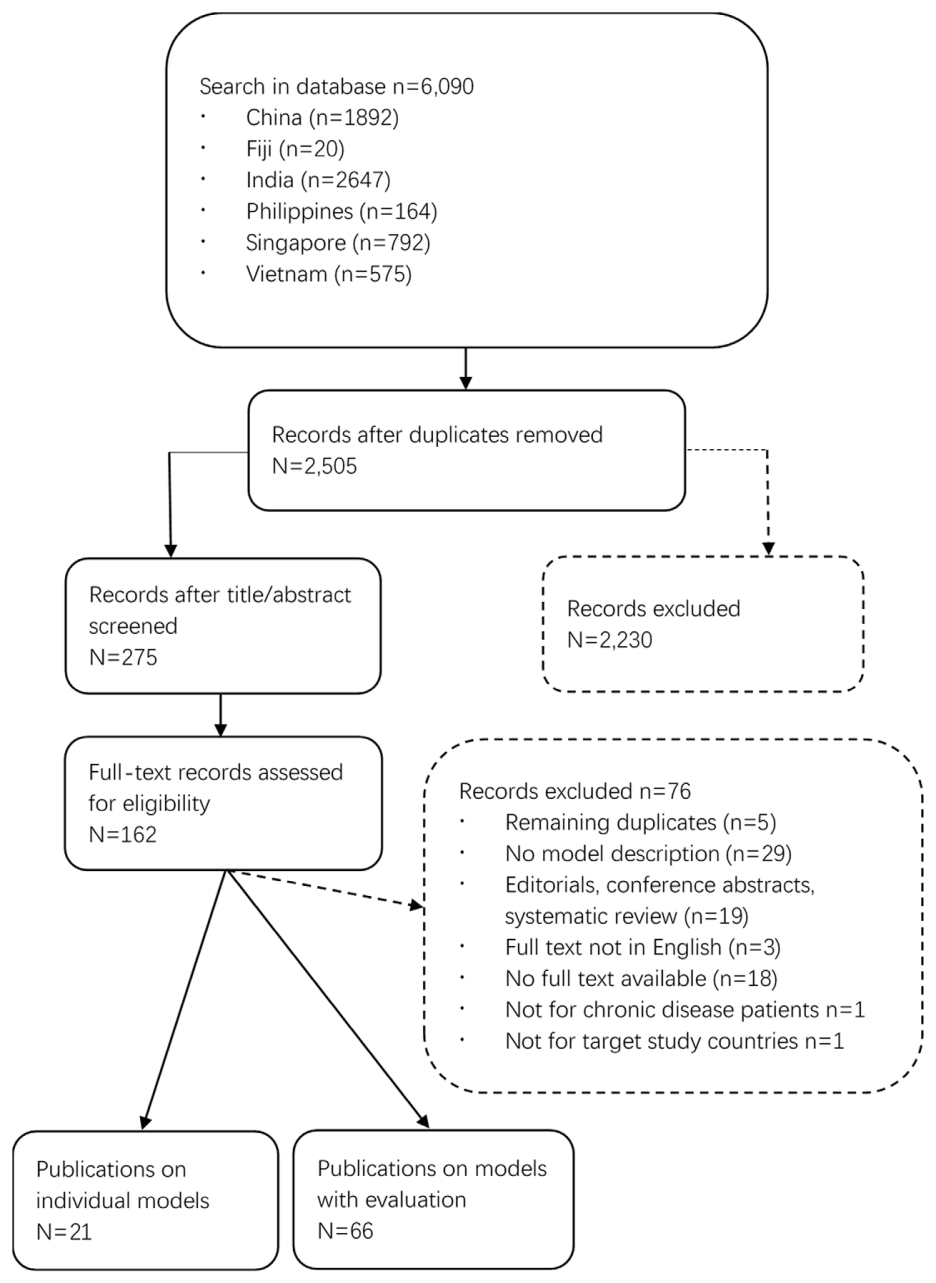
Flow chart of literature search.

The literature search is summarized in ***Figure 1***. The search yielded 6,090 potentially relevant publications. Duplicates were removed, and the remaining 2,505 publications were screened based on the title and abstract. During this stage, 2,230 articles were excluded and the remaining further 275 articles were selected for in-depth full text screening. In this phase, articles were excluded because they did not include a model description, were not chronic disease focused, were editorials, conference abstracts, systematic reviews or a full text was not available. This in-depth screening process resulted in 116 unique publications for inclusion in the data extraction process.

From the articles identified, 19 were qualitative studies, 19 were a programme or framework description, 35 were randomized studies and 17 were observational studies. Scope of implementation, breadth and degree of integration, programme attribute and target conditions are presented in ***Table 2***. A detailed table of integrated care programmes, by country, is shown in the Appendix.

**Table 2:** Basic characteristics of integrated care programmes.


	PROGRAMME CHARACTERISTIC	TOTAL (=87)	COUNTRY (N=)					

			CHINA (=44)	INDIA (=12)	SINGAPORE (=21)	FIJI (=1)	VIETNAM (=5)	PHILIPPINES (=4)

Scope of Implementation	National	21	2	12	5	1	1	/

	Regional	63	41	/	14	/	4	4

	Unknown	3	1	/	2	/	/	/

Breadth of Integration	Horizontal	13	6	2	1	/	3	1

	Vertical	31	20	1	6	/	1	3

	Both	23	5	9	7	1	1	/

	Unknown	20	13	/	7	/	/	/

Degree of Integration	Full	6	/	3	1	/	/	2

	Coordination	29	16	2	9	/	1	1

	Linkage	22	13	3	2	/	3	1

	Coordination & Linkage	6	/	4	1	/	1	/

	Unknown	24	15	/	8	1	/	/

Programme Attribute	Public	62	36	2	15	1	5	3

	Private	3	2	1	/	/	/	/

	Public & Private	19	5	9	4	/	/	1

	Unknown	3	1	/	2	/	/	/

Target Condition	General NCDs	25	14	2	8	1	/	/

	Individual Diseases	52	26	5	12	/	5	4

	Multiple Diseases	10	4	5	1	/	/	/


### Programme type and characteristics

The type of care integration within a programme is defined by the breadth of integration (horizontal, vertical or both) and the degree of integration (full integration, coordination and linkage). Out of all programmes identified, the breadth of integration was vertical for 31 programmes, horizontal for 13, both vertical and horizontal for 23 and unknown for 20. Regarding the degree of integration, 6 of the programmes were fully integrated, 29 were coordinated, 22 described a linkage, and the degree of integration was unknown for 24.

The scope of the implementation of the programmes varied, with 21 having a national scope, 63 regional and 3 programmes unknown. Most programmes (n = 62) were public, only 3 were private and 19 programmes had both public and private attributes. 25 programmes targeted the general population with non-communicable diseases, 52 targeted patients with individual diseases, and 10 targeted those with multiple diseases.

### Targeted chronic diseases/conditions by country

Out of the total number of integrated care programmes for chronic diseases, China had the highest number of programmes found in the literature (n = 44). 21 programmes were identified in Singapore, 12 in India, 5 in Vietnam, 4 in the Philippines and only one programme was found in Fiji. The eCROPS integrated care programme in China, the Integrated care pathway (ICP) programme in Singapore and the Singapore Programme for Integrated Care for the Elderly (SPICE) were described in more than one publication. Other programmes in all other countries were only described in single publications. In most study countries including China, India, Singapore and Fiji the number of integrated care programmes addressing general NCDs was higher than the number of programmes addressing any other individual or combined diseases. ***Table 3*** presents the diseases targeted by the integrated care programmes for chronic diseases by country.

**Table 3:** Number of integrated care programmes by country and disease.


DISEASE	COUNTRY (N=)					

	CHINA (=44)	INDIA (=12)	SINGAPORE (=21)	FIJI (=1)	VIETNAM (=5)	PHILIPPINES (=4)

General NCD Population	14	2	8	1	/	/

Diabetes	5	/	3	/	/	2

Tuberculosis	3	/	/	/	/	1

Hip Fractures	/	/	3	/	/	/

COPD	3	/	2	/	/	/

Hypertension	3	/	/	/	/	/

Cancer	2	2	/	/	/	/

Dementia	/	/	2	/	/	/

HIV	4	1	/	/	4	/

STD	1	/	/	/	/	/

End-stage renal failure	1	/	/	/	/	/

Pre-term infants	1	/	/	/	/	/

CVD	1	/	/	/	/	/

Mental-health disorders	2	1	/	/	1	1

Coronary health disease	/	1	/	/	/	/

Cardio-metabolic syndrome	1	/	/	/	/	/

Rheumatic diseases	/	/	1	/	/	/

Osteoporosis	/	/	1	/	/	/

Palliative Care	/	/	1	/	/	/

Multiple above conditions	4	5	/	/	/	/


### Elements of integrated care delivery

***Table 4*** shows a summary of the elements of integrated care for chronic diseases by country, divided according to three dimensions of integration adapted from the CCM: delivery system design, clinical information system and self-management support.

**Table 4:** Summary of the elements of integrated care found in the models identified from the literature.


DIMENSIONS OF INTEGRATION	ELEMENTS OF INTEGRATION	TOTAL (=87)	COUNTRY (N=)					

			CHINA (=44)	INDIA (=12)	SINGAPORE (=21)	FIJI (=1)	VIETNAM (=5)	PHILIPPINES (=4)

**Delivery System Design**	Multidisciplinary team	63	26	12	19	/	2	4
	Care coordinator/care manager	43	18	5	14	/	3	3
	Referral system	30	16	2	8	/	1	3
	Defined eligibility criteria	33	11	8	9	/	2	3
	Risk stratification of patients	19	8	4	5	/	/	2
	Single assessment	11	/	3	6	/	2	/
	Formulation of health plan	35	14	6	12	/	/	3

**Clinical Information System**	Information sharing system	33	20	5	6	/	/	2
	Use of telehealth	28	15	2	9	/	1	1

**Self-Management Support**	Engaging users	34	17	1	12	/	2	2
	Self-management support	42	22	5	12	/	/	3
	Support of informal carers	17	5	5	3	/	2	2


While all the integrated care programmes for chronic diseases included in this paper focus on clinical care, many also include broader and non-clinical elements of public health, namely health promotion and disease prevention activities. All three dimensions of care integration can include elements of public health. Delivery system design includes lifestyle coaching provided to patients by healthcare providers along with educational materials to promote their health and well-being. Within clinical information systems, telehealth is often used to facilitate lifestyle changes and provide patients with motivation. Finally, in some programmes both patients and informal carers receive coaching to control the patients’ condition and prevent future episodes.

#### Delivery system design

The majority of integrated care elements identified within the programmes, fall under delivery system design (***Table 4***). Since a healthcare delivery system includes service provider personnel, some service delivery elements of care integration are directly related to professional care providers. A multidisciplinary team reflects the integration of various service providers and the care coordinator or case manager is responsible for patient support, formulating a patient care plan and ensuring that the transfer of patients between care settings occurs smoothly and efficiently. From the programmes identified, 63 programmes described the involvement of a multidisciplinary team and 43 mentioned the involvement of a care coordinator or case manager. Other elements of delivery system design include the existence of a referral system, defined eligibility criteria for the patient population, risk stratification of patients, whether patients undergo a single assessment or have follow-up assessments and the formulation of a health plan. 30 of the programmes were found to have a referral system with a single point of referral, 33 outlined eligibility criteria for patients, 19 described risk stratification of patients, 8 had a single point of patient referral, 8 included only a single assessment and 35 programmes involved the formulation of a health plan.

A multidisciplinary team often means that clinical specialists work together with primary care physicians or clinical care teams work with social care teams or other patient support. The care coordinator ensures the seamless transfer of patients between care settings. A referral system selects patients into the care programme based on the targeted chronic diseases. Risk stratification is important for separating patients based on likelihood of certain health outcomes and thus predicting use of health services and need for multiple assessments [18].

#### Clinical Information System

Another dimension of delivery of integrated care is clinical information systems which can be further broken down and described as the use of shared electronic medical records and the use of telehealth (***Table 4***). 33 programmes used an information sharing system and 28 used telehealth. These elements of clinical information systems were often found to facilitate other elements of delivery system design such as multidisciplinary teams and care planning as well as self-management support. Many programmes make use of an information sharing system in the form of electronic healthcare data or a customized IT system storing patient records. Telehealth platforms were used in some programmes for engaging and monitoring patients, and as an educational and data sharing platform.

#### Self-management support

Healthcare is also integrated on a personal level, defined by directly engaging health service users, through self-management and informal and/or community care support (***Table 4***). 34 programmes reported engaging users, 42 provided self-management support and 17 supported informal carers. Self-management support is often enabled by other elements of integration such as telehealth which also acts as an educational platform. Patients are often provided with counselling, coaching and education as well as incentives such as gifts to support self-management. Focus on supporting informal carers involves family members and/or communities in patient care through education, training programmes and/or community mobilization.

#### Incentives and facilitators for integrated care programmes on chronic diseases

Out of 87 programmes reviewed, 38 identified facilitators for integrated care programmes for chronic diseases. Only 26 described financial incentives, some for the patients, some for the provider and a few for both. Payment systems that incorporate financial incentives are key for encouraging providers to participate in and implement integrated care programmes for chronic diseases. Performance-based incentives provide additional payments to participating providers such as pay-for-performance schemes. Innovative payment models such as bundled payment schemes or gain-sharing also encourage care providers to achieve improved value for money [19,20,21]. Financial incentives encouraging patients to enroll in integrated care programmes include reduced or waived copayments or a personal health budget providing patients with either cash or vouchers to purchase home-based care services [22]. Although none of the models described a personal health budget or direct financial contributions to service users, subsidies for medicines and services were more common. Non-financial incentives for patients included rewards for desired behavioural change and vouchers for services performed within a specific programme [23,24]. Non-financial incentives for care providers were also identified, for example giving out awards and memberships in an integrated care network as a reward for physicians and healthcare workers participating in an integrated care programme [19,24].

Facilitators of integration at the system level consist of rules and policies that facilitate an environment promoting the integration of care and making integration possible. Strong leadership and political commitment as well as community engagement acted as strong facilitators of the programmes. Many programmes function under national guidelines and frameworks of care integration which facilitate the engagement of care professionals as well as leadership and strengthen the credibility of the programme [25].

### Barriers to integrating care

Barriers were found on multiple fronts, for users, providers and the broader environment or community where integration was taking place. Some system barriers identified included a lack of supporting policies or contradictions between policies on different levels and lack of commitment in government and/or local administration. Stigma in the community and instability such as regular displacement of patients or conflict in the region was also found to undermine the success of care integration. Financial barriers and/or a lack of financial incentives affected the participation of both users and providers in integrated care programmes. Additional barriers relating to providers also included a shortage of professionals and a lack of training, expertise and/or mentorship. A significant barrier for patients was a lack of engagement within the programmes. These barriers threaten not just the implementation but also the sustainability of newly implemented care integration programmes.

### Performance assessment of integrated care programmes

It is vitally important to assess the performance of integrated healthcare programmes/models for chronic diseases. Developing a list of indicators for the performance assessment might be the first step to understanding whether these integrated care programmes have achieved the objectives intended. We have summarized indicators related to the structure, process and outcome of the programmes, which we found in the literature and presented these in ***Table 5***. The performance of the structure of the integrated care programmes was mainly assessed based on the proportion of specialists to other doctors, the sharing of medical records between hospitals and other care providers as well as access to medical technology. The performance of the process of the integrated care programmes was assessed based on access to health care (ie. convenience of care utilization, patient waiting time, patient health-seeking pathway etc.), hospital and A&E attendances, patient transfers between care providers and care settings, personalized care planning, management of medications and coordination of primary care with other health care. The performance of the integrated programmes outcome was assessed based on the number of hospital readmissions, care utilization (ie. Hospital utilization, social care utilization), quality of life as reported by patient and/or carer, ability to live independently, ability to self-manage condition, number of adverse health events, patient and/or carer reported satisfaction, transitions in care delivery (ie. Gaps in scheduled care, clear process when moving between care providers, information sharing between care providers etc.), total cost of care and clinical outcome (ie. Mortality, rate of complications etc).

**Table 5:** The performance of the integrated care programmes measured by Donabedian’s framework for healthcare quality and chosen indicators.


PERFORMANCE	INDICATORS	N=

Structure	Proportion of specialists to other doctors	1
	Sharing of medical records	1
	Access to medical technology	1

Process	Access to healthcare	25
	Coordination of primary care with other care	6
	Hospital and A&E attendance	3
	Transferring between care providers and care delivery settings	1
	Personalized care planning	1
	Management of medications	0

Outcome	Clinical outcomes	26
	Patient and carer reported satisfaction	19
	Care utilization	18
	Quality of life	17
	Total cost of care	12
	Ability to self-manage condition	6
	Number of hospital readmissions	6
	Transitions in care delivery	1
	Number of adverse health events	0
	Ability to live independently	0


Indicators used to evaluate the performance of integrated care programmes for chronic diseases were rarely applied by these integrated care programmes. Overall, only 3 programmes were found to mention indicators related to the performance of the structure of the models. Access to healthcare was the indicator most often used to evaluate the process of the models and clinical outcomes was the indicator most often used to evaluate the outcomes of the models. Care utilization, quality of life, care satisfaction and total cost of care were also frequently used to evaluate outcomes. From the indicators evaluating the performance it can be seen overall, that integrated care programmes increase access to healthcare, improve clinical outcomes and patient satisfaction. Some programmes also showed improved performance in areas of public health, such as reporting improved patient knowledge and compliance.

## Discussion

Integrated care is one strategy for achieving universal health coverage (UHC) and sustaining UHC in the face of the growing need for long-term and complex care [8]. Understanding the mechanisms and elements of integrated care programmes in the context of these diverse countries is important for drawing lessons for the future. The programmes which have been identified, range from those in an early development phase such as a community health center-led integrated care pilot for NCDs in Huangzhou, China to more developed programmes which have been nationally implemented such as the Aged Care Transition (ACTION) Program in Singapore [26,27]. The programmes also vary in the level of integration ranging from linkages to fully developed integrated care programmes. Since there is much variation between the settings, target diseases and populations as well as specific interventions, at this point it is not possible to draw firm conclusions regarding the effectiveness of the programmes. However, one can already make some observations regarding the general facilitators and barriers of care integration as well as the level of evaluation of the programmes.

The frameworks for analyzing integrated care fall into two categories: the framework based on the Valentijn model, and the framework based on the WHO framework. The Valentijn model describes six dimensions of integrated care including (1) systemic integration, (2) organisational integration, (3) functional integration, (4) professional integration, (5) service integration, and (6) normative integration. In this framework, systemic integration reveals the macro level of the integrated care, professional and organisational integration illustrates the meso level of the integrated care, clinical integration describes the micro level of the integrated care. Functional and normative integration play the role of enablers to connect the different levels with each other [48]. Alternatively, the WHO framework analyzes care integration using five key elements including (1) empowering individuals and communities through health education, knowledge sharing and training programmes, (2) strengthening participatory governance and mutual accountability, (3) improving health service delivery by prioritizing primary care outpatient and ambulatory care, strengthening population health and enabling new technologies, (4) coordinating care from different levels for people, making connections and communication between health providers and (5) creating a favorable environment through improving management, upgrading the information system and optimizing the incentive mechanism for health providers [6].

To date, integrated care has been more commonly implemented in western countries, however the concept has been gaining popularity and piloted in Asia-Pacific region also, [5] and therefore many of the programmes and studies in the Asia-Pacific region are reflections of those in western countries. As the Asia-Pacific has a huge ageing population across diverse settings with a rise in those requiring care for complex and chronic conditions, there are many lessons to be learned from understanding which features of integrated care programmes are contributing to their success [11]. As the settings in Asia-Pacific vary vastly from highly developed urban Singapore to less-developed rural India, we can understand how elements of integration are adapted to a variety of settings and have an idea of which elements are the most important in all settings.

Although there is no single approach or model which best supports integrated care, there are several factors which contribute to the success of integrated care programmes [28]. Facilitators and barriers can be categorized according to external context (laws, regulations, an already existing health system, strategic direction), system organization (financing, organizational leadership, structure of existing services, culture), intervention organization (intervention size and complexity, resources, credibility), as well as providers and research staff (shared values, engagement, communication) [26]. The particular factors influencing the success of a programme varies according to the context. A 2015 systematic review of the facilitators and barriers of implementing chronic care models in countries across North America and Europe found that the strongest general facilitators included strong networks and communication, a culture and implementation climate which supported integration and uptake of chronic care, strong leadership, provider knowledge and beliefs about the programmes to be implemented [29]. General barriers in these settings were related to the execution of integrated care programmes, lack of organizational readiness, no support and accountability from senior leadership as well as a negative attitude and a lack of buy-in from care providers [28].

In comparison, across countries in Asia-Pacific region many barriers arise from health system instability and a lack of information management stemming from inadequate IT infrastructure and low resources [5]. Integrated care programmes can address some of these obstacles to provide a continuum of care for chronic conditions. For example, as integrated care emphasizes information sharing, it can be used to facilitate communication between providers and patients as well as within multidisciplinary teams.

Care integration aims to reduce overlap between services and improve coordination of care between professionals thus improving cost effectiveness, although it must be noted that integration does not solve a lack of resources [5,30]. An important difference between integrated care programmes in western countries and the Asia-Pacific is the care coordinator. A review of seven integrated care programmes in western countries described care coordinators as the distinguishing feature contributing to the success of all the programmes and also found the engagement of patients and their communities to be essential [31]. Our review in comparison has found self-management support to be present in less than half the programmes and care coordinators were also not present in every programme, although this may be attributable to a lack of human resources and/or culture in some settings [11,32].

In our review, the enabling factors varied by specific context and study country. Many of the programmes identified in India clearly mentioned the success of programmes rested upon adapting them to the local context to ensure acceptability with the local staff and patients [33,34,35]. In Singapore, strong government involvement and leadership was frequently cited as a facilitator [36,37,38]. Government support was also identified as a facilitator in the Philippines along with strong community involvement [39,40]. Facilitators of integrated care programmes in China included community involvement, government support and existence of national guidelines [21,25,41,42]. Clearly strong leadership and a supportive setting is essential and can influence the success of integrated care programmes [43]. The role of technology and sophisticated IT systems in integrating healthcare is also necessary for managing and sharing patient or service-related information. However, IT literacy is inconsistent across Asia-Pacific and electronic medical records are not the norm in all healthcare settings in the region [6].

Various payment methods have an important role in encouraging patient participation, clinical guideline maintenance and treatment adherence, in addition to achieving health targets [22]. These varying payment methods have offered financial incentives to support the structure, process and the outcome elements of integrated care models [22]. In many western countries financial incentives for integration of care most often target providers, but some also focus on health insurers and patients [22,44]. We have found that the financial performance of integrated care programmes in the literature was not frequently evaluated. Cost-effectiveness was discussed in very few programmes in India, Singapore and China [27,37,38,45]. These programmes cited lowering of costs by reduced hospitalization and increased efficiency of services.

A monitoring and evaluation mechanism to provide feedback is also important for identifying potential issues and informing programme leaders and policy makers, however these were not often done adequately based on the literature we have reviewed. The goals of integration as defined by the WHO is to enhance the quality of care and quality of life, increase consumer satisfaction and increase system efficiency [46]. Performance evaluation should measure the degree to which these goals are met. A systematic review of integrated care models in the UK and abroad, found that the three most frequent indicators of care integration which provide the strongest measure of effect included higher patient satisfaction, increased perceived quality of care and increased patient access to services [47]. In accordance with these findings, this review also found that improved access to healthcare and increased patient satisfaction were the most frequently cited performance indicators. Overall, considering the growing burden on healthcare systems by several factors associated with aging, increasing prevalence of NCDs and emerging stress factors associated with changing lifestyles or environmental conditions, the need for integrated care is likely to increase.

## Strengths, challenges and limitations

One of the challenges of investigating integrated care is the lack of clear definitions and boundaries for this term. The search terms were defined in a way to retrieve all relevant models for this study, however some may have been missed, due to the various terms associated with integrated care which were not included in the search terms. There is also no statistical analysis in this review, because of the variation in the models. It would be a formidable challenge to identify any sort of causal relationship between the models of integrated care and the impact on the delivery of care and care outcomes [47]. This review paper does not attempt to draw any causal conclusions per se. We have also not completed a critical appraisal of the studies included, as that would be outside the scope of a scoping review. Another limitation is that among the papers we reviewed, some care integration “models” were actually pilots or examples of specific programmes not new models, and therefore since these did not provide an overview of the type of full models we intended to examine we excluded these. Finally, the scoping review only covered academic publications. Those reported by any grey literature have been left out, which could have led to some bias since studies with neutral, null or negative findings are less likely to be published [49]. The strength of this scoping review is that to our knowledge, no other literature exists which summarizes the elements, processes and outcomes of integrated care in the Asia-Pacific region.

## Conclusion

Healthcare integration is increasingly emerging as a response to the challenges requiring delivery of long-term healthcare services. Although the definition and forms of integrated care are various, the increasing trend of integrated care programmes has been recognized in the Asia-Pacific countries. Integrated care seeks to improve healthcare delivery systems to ensure that patients receive appropriate, equitable and affordable healthcare services. However, many studies do not have a rigorous performance evaluation for emerging pilot integrated care programmes. For better understanding the value of integrated care and developing strategies for implementation, more performance assessment of integrated care programmes is essential.

## Additional File

The additional file for this article can be found as follows:

10.5334/ijic.5439.s1 Appendix 1. Definitions of features of integrated care programs and associated terms [35,36,33].
