# Supplementary material for: Elements and Performance Indicators of Integrated Healthcare Programmes on Chronic Diseases in Six Countries in the Asia-Pacific Region: A Scoping Review
Source: Int J Integr Care. 2021 Feb 8;21(1):3. doi: 10.5334/ijic.5439 (PMC7879996; doi:10.5334/ijic.5439)
Supplement: Appendix 1. — Definitions of features of integrated care programs and associated terms [353633]. [file ijic-21-1-5439-s1.pdf]

(9.) Appendix

Appendix 1 Definitions of features of integrated care programs and associated terms [35, 36, 33]

Term	Definition
Patient-centered care	An approach to care that consciously adopts individuals', carers', families' and communities' perspectives as participants in, and beneficiaries of, trusted health systems that are organized around the comprehensive needs of people rather than individual diseases, and respects social preferences
Coordinated care	Linking of health care events and services so that the patient receives appropriate care for all his/her health problems, physical as well as mental and social
Systemic integration	Refers to the alignment of rules and policies within a system
Organizational integration	Refers to the extent to which organizations coordinate services across different organizations
Horizontal integration	Relates to strategies that link similar levels of care
Vertical Integration	Relates to strategies that link different levels of care
Full Integration	Refers to formally pooling resources, allowing a new organization to be created alongside development of comprehensive services attuned to the needs of specific patient groups
Coordination	Refers to operating through existing organizational units so as to coordinate different health services, share clinical information and manage transition of patients between different units
Linkage	Takes place between existing organizational units with a view to referring patients to the right unit at the right time, and facilitating communication between professionals involved in order to promote continuity of care. Responsibilities are clearly aligned to different groups with no cost shifting
Functional Integration	Refers to the extent to which back-office and support functions are coordinated
Professional Integration	Refers to the extent to which professionals coordinate services across various disciplines
Service Integration	Refers to coordination across commissioners and providers and typically focused on breaking down barriers across service providers
Personal Integration	Refers to involving service users and their informal carers or family members into the decision-making process
Normative Integration	Refers to the extent to which mission, work values etc. are shared within a system

Appendix

Program name	Author, Year, Country	Target condition	Implementation scope	Main sectors	Model type	Delivery System Design	Clinical Information System	Self-Management Support	Incentives	Achievements (results of performance evaluation)
eCROPS	Rui Feng et al., 2013, China	Diabetes	Regional	Public	NA	Care coordinator, risk stratification of patients	Use of telehealth	Self-management support	Diabetes prevention is financially reimbursed to the doctor based upon service volume and quality	Introduced a package of long-term incentives, established ongoing mechanisms for continuous capacity building and quality improvement, and built up an operational cycle for catalyzing similar efforts.
eCROPS-CA	Jing Chai et al., 2015, China	Cancer	Regional	Public	NA	Care coordinator, risk stratification of patients	Use of telehealth	Self-management support	Participating doctors meeting set performance standard get reimbursed at about $3.5 per case-year	
IMPACT	Weibin Cheng et al., 2016, China	HIV	Regional	Public	NA	Risk stratification of patients	Use of telehealth	Self-management support		Enhanced awareness, service uptake, retention in care and adherence to treatment. Increased access to HIV related services.
The Chinese Older Adult Collaborations in Health (COACH)	Shulin Chen et al., 2018, China	Comorbid depression and hypertension	Regional	Public	Horizonal, linkage	Multidisciplinary team	Use of telehealth	NA	PCPs, AWs, and Psychiatrists in the COACH study arm and PCPs in eCAU villages receive a small salary stipend from the Departments of Health of Tonglu and Jiande counties for their added effort in support of the study	Improved health outcomes.
LEAN	Dong (Roman) Xu et al., 2016, China	Schizophrenia	Regional	Public	Horizonal, linkage	Multidisciplinary team, care coordinator, risk stratification of	Use of telehealth	Engaging users,	Patients and LHSs will accumulate points for	

						patients		Self-management support, Support of informal carers	responding to SMS messages. Each of their texted confirmations back to the LEAN system will accumulate one point, which will be recorded automatically by the computer system. The points, counted every 2 months, will advance their Taekwondo-like belt ranking and entitle them to a small gift of US$2–3 such as soap bars	
programme on glycemic control and behavioral outcomes for type 2 diabetics	Xiaodan Yuana et al., 2016, China	Type 2 diabetes	N/A	Private	NA	Multidisciplinary team, care coordinator, formulation of health plan, single point of patient referral	NA	Engaging users,<br><br>Self-management support		Improved health outcomes.
Taiyuan Central Hospital medical consortium	G Shi et al., 2012, China	cancer	Regional	Public	Vertical, linkage	Risk stratification of patients, defined eligibility criteria<br><br>single point of patient referral	NA	NA		Reduced length of stay and total hospitalization costs.
Integrated TB service model	Xiaolin Wei et al., 2013, China	Tuberculosis	Regional	Public	Vertical and horizontal, linkage	Defined eligibility criteria	NA	NA	financial support from Global Fund in poorer western provinces	Shorter delays for patients.
Integrated PHSHT services	Jianhong Xia et al., 2015, China	HIV	Regional	Public	Vertical and horizontal, linkage	Multidisciplinary team	NA	Engaging users,<br><br>Self-management support		
integrated TB control model	Xiaolin Wei et al., 2013, China	Tuberculosis	Regional	Public	Vertical and horizontal, coordination	Risk stratification of patients	NA	NA		Lower number of providers visited, shorter treatment delays, lower medical expenditure.
PCP-Cardiologist Telemedicine Model (PCTM)	Lei Xu et al., 2017, China	hypertension	Regional	Public	Vertical, coordination	Multidisciplinary team, risk stratification of patients, single point of patient	Use of shared electronic healthcare data, use of	Engaging users,<br><br>Self-		Improved health outcomes.

						referral	telehealth	management support		
patient-centered cognitive behavioral therapy	Ying Zhang et al., 2016, China	cardio-metabolic syndrome	Regional	Public	NA	Multidisciplinary team, care coordinator, formulation of health plan	NA	Engaging users,<br><br>Self-management support		Improved health outcomes.
nurse-led telephone support model	Juan Li et al., 2014, China	end-stage renal failure	Regional	Public	NA	Care coordinator, single point of patient referral, formulation of health plan	Use of telehealth	Engaging users,<br><br>Self-management support, Support of informal carers		Improved health outcomes and patient satisfaction.
intergrative strategy of health service delivery for rural hypertension patients	Yudong Miao et al., 2016, China	hypertension	Regional	Public	Vertical, coordination	Multidisciplinary team, care coordinator, defined eligibility criteria, single point of patient referral	Use of shared medical record	Self-management support		Improved health outcomes and quality of life.
a comprehensive intervention project in Qianjiang District	Wenxi Tang et al., 2015, China	Hypertension and/or type 2 diabetes	Regional	Public	Vertical and horizontal, coordination	Multidisciplinary team, care coordinator, single point of patient referral	Use of shared medical record	NA	System global budgets and pay-for-performance (SGB and P4P) paid by medical insurance	
home-based physiological information acquisition system	Yan Yan et al., 2013, China	General NCDs	Regional	Private	NA	Multidisciplinary team	Use of shared medical record	Engaging users,<br><br>Self-management		Improved response time.
the medical-nursing combined care	J Bao et al., 2015, China	General NCDs	Regional	Public & Private	Horizontal, coordination	Care coordinator	NA	NA		
A community based integrated intervention for early prevention and management of COPD	Yumin Zhou et al., 2010, China	COPD	Regional	Public	NA	Use of risk stratification	Use of telehealth	NA		Improved prevention and management of disease.
The public CHC model	Leiyu Shi et al., 2015, China	General NCDs	Regional	Public	NA	NA	Use of shared medical record	NA		Improved quality and value of care.
The gate-keeper	Leiyu Shi et al., 2015,	General NCDs	Regional	Public	NA	Care coordinator	Use of shared medical	NA		Improved quality and value of

CHC model	China						record			care.
The hospital owned CHC model	Leiyu Shi et al., 2015, China	General NCDs	Regional	Public	Vertical	Single point of patient referral	Use of shared medical record, use of telehealth	NA		Enhanced accessibility and continuity of care. Improved quality and value of care.
GPs taskforce and contract-based care delivery	Yue Xiao, 2015, China	General NCDs	Regional	Public	Vertical, linkage	Single point of patient referral	Use of shared medical record, use of telehealth			
vertical integration of NCDs care	Yue Xiao, 2015, China	General NCDs	Regional	Public	Vertical, coordination	Multidisciplinary team, single point of patient referral	Use of shared medical record, use of telehealth	NA	funding support from the provincial, municipal, and county governments	
'1+1+1' model	Yue Xiao, 2015, China	General NCDs	Regional	Public	Vertical, coordination	Multidisciplinary team, care coordinator, single point of patient referral	Use of shared medical record, use of telehealth	NA	Implementing a zero drug markup policy, abolishing earmarked subsidies for outpatient care in tertiary hospitals, and setting up performance assessment targets for clinical integration with community centers; Patients are getting public subsidies, claiming higher reimbursements, and making direct appointments with specialist online	
Domiciliary Integrated pulmonary rehabilitation (PR) Program	Yi Li et al., 2018, China	COPD	Regional	Public	Vertical, coordination	Multidisciplinary team, defined eligibility criteria, formulation of health plan	NA	Engaging users,<br><br>Self-management support		Improved clinical performance, reduced emergency department visits and reduced episodes of hospitalization.
Community-based intervention packages	Zohra S et al., 2015, China	Other	Regional	Public	Vertical, coordination	Multidisciplinary team, defined eligibility criteria, formulation of health plan	NA	Engaging users, Support of informal carers	A global budget for a single hospital plus flatrate case payment	Improved healthcare related outcomes.
an initiative to promote an elder-friendly Hong	Jean Woo, 2017, China	General NCDs	Regional	Public	Vertical, coordination	Multidisciplinary team, formulation	NA	Engaging users,	Expenditures are partly covered by	

Kong						of health plan		Self-management support	government	
Integrated hospital-community diabetes management program	Siyu Chen et al., 2017, China	Diabetes	Regional	Public	Vertical, linkage	Multidisciplinary team, care coordinator, single point of patient referral, formulation of health plan	Use of shared medical record	NA		Improved health outcomes.
Joint Asia Diabetes Evaluation (JADE) program	Juliana C.N. Chana et al., 2014, China	Diabetes	Regional	Public & Private	Horizontal, coordination	Multidisciplinary team, care coordinator, single point of patient referral, defined eligibility criteria, formulation of health plan	Use of shared medical record	Self-management support		Increased accessibility, affordability and sustainability.
City-driven prevention of mother-to-child transmission (PMTCT) program	Song, Junmin et al., 2013, China	HIV	Regional	Public & Private	Horizontal, linkage	Multidisciplinary team, defined eligibility criteria, formulation of health plan	NA	Self-management support		Increased access to antiretroviral prophylaxis.
Integrated care and discharge support for elderly patients (ICDS)	Francis OY Lin et al., 2015, China	General NCDs	Regional	Public	Vertical, linkage	Multidisciplinary team, care coordinator, defined eligibility criteria, formulation of health plan	Use of shared medical record	Engaging users, Support of informal carers	Programme is government funded	Reduced accident and emergency department attendance, acute hospital admissions and hospital bed days.
Integrated care pilot	Yi Qian et al., 2017, China	General NCDs	Regional	Public	Vertical, coordination	Multidisciplinary team, Single point of patient referral, Defined eligibility criteria, Formulation of health plan	Use of shared medical record	NA		Community health center was able to act as gatekeeper.
Integrated medical rehabilitation delivery	Yue Xiao et al., 2017, China	General NCDs	Regional	Public & Private	Vertical, linkage	Multidisciplinary team, Defined eligibility criteria, Formulation of health plan	NA	NA	Drug share of total revenue decreased, leading to decrease in out of pocket payments	
integrated intervention for prevention and management of COPD	X. Yuan et al., 2015, China	COPD	Regional	Public & Private	Vertical, linkage	Multidisciplinary team, care coordinator	NA	Engaging users,<br><br>Self-management support		Improved health outcomes and lower death rate.

the model of vertical integrated care between the three-levels of healthcare institutions	Shaofan Chen et al., 2018, China	Type 2 diabetes	Regional	Public	Vertical, coordination	Multidisciplinary team, care coordinator	Use of shared medical record	Engaging users,<br><br>Self-management support		Improved patient care, satisfaction and self-management. Improved health care worker knowledge and improved quality of care provided.
Integrated care model for patients with kidney diseases	Xiaohui Zhang et al., 2014, China	Kidney diseases	Regional	Public	Vertical, coordination	NA	Use of shared medical record, use of telehealth	Engaging users,<br><br>Self-management support	The treatment cost of local rural residents is paid for primarily by the rural cooperative medical insurance, other medical insurances, or privately	Improved health outcomes and patient survival.
Care System integration in rural China	Xin Wang et al., 2016, China	General NCDs	Regional	Public	vertical	Single point of patient referral, Formulation of health plan	Use of shared medical record,	NA		Improved collaboration between care provision institutions.
hypertension management trial in rural China	Yuting Zhang et al., 2017, China	hypertension	Regional	Public	Vertical, coordination	Multidisciplinary team, care coordinator, Single point of patient referral	Use of shared medical record	Self-management support	If at the end of the performance year, the total actual in- patient spending for all patients with hypertension in Group 2 towns was above the benchmark amount, the providers who participated in the trial would be paid according to the regular reimbursement schemes; however, if the total actual inpatient spending was below the predicted benchmark amount, they would obtain a bonus at 60% of total savings	Improved health outcomes and quality of life. Reduced rates of hospitalization.
integrated approach for tuberculosis	Qiang Sun et al., 2012, China	tuberculosis	Regional	Public	Vertical, linkage	Single point of patient referral, Formulation of health plan	NA	Engaging users,<br><br>Self-management support		High treatment success rate, lower medical expenditure and shorter health system delay.

integrated health management model	Jianqian Chao et al., 2013, China	General NCDs	Regional	Public	NA	Engaging users,<br><br>Self-management support	Use of telehealth	NA		Improved satisfaction.
Integrated PMTCT Service	Ai-Ling Wang et al., 2015, China	HIV	National	Public	Horizontal, linkage	Multidisciplinary team	Use of shared medical record	NA	Financially supported by the Government of China. From 2010 to 2013, the central government allocated over 3.4 billion Yuan (573 million USD), to implement these services.	
Family Integrated Care (FIC)	Shi-wen He et al, 2018, China	other	Regional	Public	NA	NA	NA	Engaging users, Support of informal carers		Improved clinical outcomes.
Integrating Depression Care in ACS patients in Low Resource Hospitals	Shenshen Li et al., 2018, China	CVD	Regional	Public	NA	Multidisciplinary team, care coordinator	Use of shared medical record, use of telehealth	Engaging users,<br><br>Self-management support		
'686 Programme' model	Di Liang et al., 2018, China	Mental disorder	National		Vertical and horizontal, coordination	Multidisciplinary team, Defined eligibility criteria	NA	NA	Government initiated funding for specialized public health projects and created an opportunity to fund the '686 Programme'	
Opportunistic screening of NCD	Amarchand R et al., 2015, India	General NCDs	National	Public	Vertical and horizontal, Coordination and linkage	Multidisciplinary team, risk stratification, defined eligibility criteria	NA	Engaging users	NA	Improved screening and utilization of services.
INDEPENDENT model	Kowalski AJ et al., 2017, India	Diabetics, depression	National	Private	Vertical and horizontal, Coordination and linkage	multidisciplinary team, care coordinator/case manager, care planning, defined eligibility criteria	NA	Self-management support	NA	
Psychosocial Intervention in Cancer Care	Turner J et al., 2011, India	cancer	National	Public & Private	Vertical and horizontal, Coordination and linkage	Multidisciplinary team, care coordinator/case manager, defined eligibility criteria, risk stratification,	Use of shared electronic data	Self-management support	No	Improved quality of life and mood. Improved symptom control, support and care coordination.

						care planning			
Provider-initiated HIV testing & counselling in incident tuberculosis cases	Mohan A et al., 2017, India	Tuberculosis and HIV	National	Public & Private	Vertical and horizontal, fully integrated	Multidisciplinary team, defined eligibility criteria, single assessment, single point of referral	use of shared electronic data	Support of informal carers	Higher proportion of TB patients underwent HIV testing.
Clinic-based multi-component CVD risk reduction intervention	CARRS Trial Writing Group et al., 2012, India	CVD and diabetes	National	Public & Private	Vertical and horizontal, Coordination and linkage	Multidisciplinary team, defined eligibility criteria, care planning, care coordinator/case manager	Use of shared electronic data, use of telehealth	Self-management support	Improved health outcomes and improved control of CVD risk factors.
Community-based intervention programmes	Krishnan A et al.,2010, India	General NCDs	National	Public & Private	horizontal, linkage	Multidisciplinary team,	NA	Self-management support, support of informal carers	Increased diagnosis and better management of NCDs at heath facilities.
Programmatic management issue solving in Diabetes mellitus and tuberculosis	Harries AD et al., 2016, India	Diabetes and tuberculosis	National	Public & Private	Vertical and horizontal, linkage	Multidisciplinary team, single assessment, defined eligibility criteria, single point of referral	NA	NA	
Integrated management of Adult Liness	R Washington et al., 2011, India	HIV	National	Public	horizontal, linkage	Multidisciplinary team, care coordinator/case manager	Use of shared medical record	Support of informal carers	Reduced loss to follow-up and decreased annual death rates. Increased quality of life and ability to cope with discrimination and stigma.
Integration of mental health in primary care	Shidhaya R., 2016, India	Mental health	National	Public & Private	Vertical and horizontal, fully integrated	Multidisciplinary team, single assessment, use of risk stratification	Use of shared medical record	Support of informal carers	Improved community mobilization. Improved awareness of mental health disorders, identification, treatment and recovery.
Lifestyle Intervention in Families for Cardiovascular risk reduction (PROLIFIC Study)	Panniyammakal Jeemon., 2017, India	Coronary heart disease (CHD)	National	Public & Private	Vertical and horizontal, fully integrated	Multidisciplinary team, care coordinator/case manager, care planning, defined eligibility criteria, use of risk	Use of telehealth	Self-management support, support of informal carers	Improved risk factor control

						stratification				
Integrated approach in improving QOL in lung cancer.	VP Shankpal., 2011, India	cancer	National	Public & Private	Vertical, coordination	Multidisciplinary team, care planning, defined eligibility criteria	NA	NA		
Private Partnership in coordinating TB and HIV.	Dholakia YN., 2012, India	Tuberculosis and HIV	National	Public & Private	Vertical and horizontal, coordination	Multidisciplinary team, care planning	NA	NA		Improved health access and quality of life of patients and peers.
Not given - however Fiji NCD plan mentioned later in the texts	Manju Rani, Sharmin Nusrat and Laura H Hawken, 2012, Fiji	NCDs	National	Public	Horizontal and vertical	N/A	N/A	N/A		
integrative and decentralized service delivery models	Bach Xuan Tran, 2015, Vietnam	HIV	Regional	Public	Horizontal, Linkage	Single assessment	N/A	Support of informal carers		Most patients prefer decentralized and integrated models
palliative care incorporated into existing HIV and cancer services	Kimberly Green,<br><br>2010, Vietnam	HIV	Regional	Public	Horizontal, Linkage	Multidisciplinary team	N/A	Support of informal carers		Improvements in quality of care
trained and mentored provincial coaching team (PCT)	Lisa A, 2015, Vietnam	HIV	Regional	Public	Vertical, Coordination	Multidisciplinary team, care coordinator/case manager, defined eligibility criteria	Use of telehealth	N/A		Successful spread of quality improvement activities, providing regular coaching for care providers
MMT/<br><br>HIV integration	Vivian F. 2016, Vietnam	HIV	National	Public	Horizontal, Linkage	Care coordinator/case manager, Single assessment	N/A	N/A		Identified barriers and programmatic challenges
Vietnam Multicomponent Collaborative Care for Depression Program	Victoria K. 2014, Vietnam	Depression	Regional	Public	Vertical and horizontal, coordination and linkage	Care coordinator/case manager, Defined eligibility criteria, single point of referral	N/A	N/A		The program was found to be acceptable, feasible and effective
No program name specified. Intervention was implemented by Medecins Sans Frontieres (MSF)	Yolanda Mueller, 2011, Philippines	Mental health disorders	Regional	Public	Vertical, coordination	Multidisciplinary team, care coordinator/case manager, Risk stratification, Single point of referral, defined eligibility criteria, care planning	N/A	Self-management support	MSF covered all transportation and psychiatric treatment costs and for referred patient for a minimum of 6 months up to two years of	After at least 2 visits, improvement in Self-Reporting Questionnaire or SRQ20 scores and Global Assessment of Functioning score (GAD) was

									treatment	seen.
First Line Diabetes Care Project (FILDCARE)	Grace Marie V., 2014, Philippines	Type 2 diabetes	Regional	Public	Vertical, fully integrated	Multidisciplinary team, care coordinator/case manager, Defined eligibility criteria, care planning Risk stratification,		Self-management support, support of informal carers		Intervention improved health knowledge among Local Government Health Unit. Improved ability to self-manage condition.
Context-adapted chronic disease-care model (CACCM)	Grace M.V. Ku, 2015, Philippines	Type 2 diabetes	Regional	Public	Horizontal, linkage	Multidisciplinary team, Single point of referral, care planning	Information sharing system	Self-management support, support of informal carers		Improved health outcomes.
the initiation of ambulatory management of drug resistant TB at the MMC	M. I. D. Quelapio, 2010, Philippines	TB	Regional	Public & Private	Vertical, fully integrated	Multidisciplinary team, care coordinator/care manager, Single point of referral, defined eligibility criteria	Information sharing system, use of telehealth	N/A	Financial support provided by Global Fund to Fight AIDS, Tuberculosis and Malaria (Global Fund)	Improved treatment success rate.
ValuedCare program	Quelapio, M. I. D 2018, Singapore	Hip fractures	Regional	Public	Vertical, coordination	Multidisciplinary team, care coordinator/care manager, Single point of referral, single assessment	Information sharing system			Improved quality and delivery of care.
Transitional care programme	Kheng Hock Lee, 2015, Singapore	General population	Regional	Public	N/A	Multidisciplinary team, Single point of referral, single assessment, care planning	Use of telehealth	Self-management support, support of informal carers		Greater reported patient satisfaction.
Delivering on Target (DOT) Programme - Diabetes	SQ Yeo, 2012, Singapore	Diabetes	National	Public & Private	Vertical and horizontal, fully integrated	Single point of referral, care planning	Information sharing system, use of telehealth		Laboratory test vouchers and subsidized drugs	Facilitated efforts to shift diabetes care to communities
integrated model of care for hip fractures	Hitendra K. 2013, Singapore	Geriatric hip fracture	Regional	Public	Horizontal and vertical, coordination and linkage	Multidisciplinary team, care coordinator/case manager, care planning	Use of telehealth	N/A		Indices like number of adverse health events and time to admission was reduced.
The Aged Care Transition (ACTION) Program	Shiou-Liang Wee 2014, Singapore	General elderly population with complex care needs	National	Public	Vertical, coordination	Multidisciplinary team, care coordinator/case manager, Defined eligibility criteria, care planning	N/A	Self-management support, support of informal carers	Funding from the Health Ministry	Unplanned rehospitalizations and ED visits after discharge reduced. Improved the transition of vulnerable older

										adults from hospital to community
Primary Care Dementia Clinic (PCDC)	Nakul Saxena, 2017, Singapore	Dementia	Regional	Public	Vertical, coordination	Multidisciplinary team Defined eligibility criteria	N/A	N/A	Funded by the Ministry of Health Community Mental Health Masterplan Program	Improved caregiver satisfaction, lower direct medical costs, higher quality adjusted life years.
Integrated practice units (IPU)	Lian Leng Low, 2017, Singapore	General population	Regional	Public	Vertical, linkage	Multidisciplinary team, care coordinator/case manager, risk stratification, single assessment, care planning, defined eligibility criteria, single point of referral	Information sharing system, use of telehealth	Self-management support		Reduced readmissions in patients who are at highest risk of readmission.
The Integrated Community of Care (ICoC)	Lian Leng Low, 2017, Singapore	General population	Regional	Public	Horizontal and vertical, coordination	Multidisciplinary team, care coordinator/case, manager, Care planning, defined eligibility criteria	Information sharing system	Self-management support		
Integrated care pathway (ICP) programme	Christine Xia Wu, 2014, Singapore	COPD	Regional	Public & Private	Horizontal and vertical and coordination	Multidisciplinary team, care coordinator/case manager, Risk stratification, care planning, single point of referral	Use of telehealth	Self-management support		
Integrated care pathway (ICP) programme	Christine Xia Wu, 2018, Singapore	COPD	Regional	Public & Private	Horizontal and vertical, coordination	Multidisciplinary team, care coordinator/case manager, Risk stratification, care planning, single assessment, defined eligibility criteria, single point of referral	N/A	Self-management support		Risk of hospitalization as well as hospital bed days was lower.
Integrated care pathway (ICP) programme	Tsung Wei Chong,<br><br>2013, Singapore	Fragility hip fractures	Regional	Public	N/A	Multidisciplinary team, care coordinator/case manager, Risk stratification, Care planning, single assessment	N/A	Self-management support		Reduced length of hospital stay.
iCommunity@East	Chui Siem, 2014,	Risk of dementia and mental	Regional	Public	Vertical,	Multidisciplinary	N/A	Self-management		Increased support for

	Singapore	disease			coordination	team		support		elderly living with dementia and their caregivers
the Singapore Programme for Integrated Care for the Elderly (SPICE)	Germaine Liu, 2015, Singapore	General elderly population	National	Public	N/A	Multidisciplinary team, care coordinator/case manager	N/A	N/A	Funded by the government	Successfully enabled seniors to age-in-place
the Singapore Programme for Integrated Care for the Elderly (SPICE)	Ho Chun Keong, Singapore	General elderly population	N/A	N/A	N/A	N/A	N/A	N/A		Lowered nursing home admission rate. Reduced acute hospital utilization. Reduced step-down care hospital utilization. Increased participant care satisfaction and reduced caregiver stress.
the National Health Group (NHG) and the Alexandra Health System (AHS)	Wee Shiong Lim , 2017, Singapore	General NCDs	Regional	Public	Horizontal and vertical, fully integrated	Multidisciplinary team, care coordinator/case manager, care planning, defined eligibility criteria, single point of referral	Use of telehealth	Self-management support	Funded by the government	Successfully enabled seniors to age-in-place
Right siting	Anita YN Lim, 2015, Singapore	Rheumatology	Regional	Public & Private	N/A	Multidisciplinary team, care coordinator/case manager, Defined eligibility criteria	N/A	N/A		Successful shared care between patients without hospital subsidies and private family physicians.
Osteoporosis Patient Targeted and Integrated Management for Active Living (OPTIMAL)	M. Chandran, 2013, Singapore	Osteoporosis	National	Public	Horizontal, coordination	Multidisciplinary team, care coordinator/case manager, Care planning, single assessment, eligibility criteria, single point of referral	Information sharing system	Self-management support	Singapore Ministry of Health-funded; funding for subsidies on anti-osteoporosis medications for certain patients	Improved compliance rate.
the Health Management Unit (HMU)	Joo Pin Foo, 2013, Singapore	Diabetes	Regional	Public	N/A	Multidisciplinary team, care coordinator/case manager, Risk stratification	Use of telehealth information sharing system	Self-management support		
The Singapore General Hospital Diabetes Centre	Emily Tse,Lin Ho, 2014, Singapore	Diabetes	Regional	Public	N/A	Multidisciplinary team	Use of telehealth			Reduced waiting times. Staff satisfaction and communication

(DBC)										improved.
the Singapore Regional Health System was introduced in the article	Milawaty Nurjono, 2016, Singapore	General population	National	Public	Vertical and horizontal	Multidisciplinary team, care coordinator/case manager	N/A	N/A		
This program does not have a name. It is referred to as a pallative program in Singapore in the article	Soek Tian Angeline, 2013, Singapore	Population requiring palliative care	N/A	N/A	Vertical, linkage	Multidisciplinary team, Defined eligibility criteria, care planning	Use of telehealth	Self-management support, support of informal carers		Reduced need for frequent hospitalizations. Satisfactory symptom control.
